# The additional value of ONEST (Observers Needed to Evaluate Subjective Tests) in assessing reproducibility of oestrogen receptor, progesterone receptor, and Ki67 classification in breast cancer

**DOI:** 10.1007/s00428-021-03172-9

**Published:** 2021-08-20

**Authors:** Bálint Cserni, Rita Bori, Erika Csörgő, Orsolya Oláh-Németh, Tamás Pancsa, Anita Sejben, István Sejben, András Vörös, Tamás Zombori, Tibor Nyári, Gábor Cserni

**Affiliations:** 1 TNG Technology Consulting GmbH, Budapest, Hungary; 2 Department of Pathology, Bács-Kiskun County Teaching Hospital, Kecskemét, Hungary; 3 Department of Pathology, University of Szeged, Szeged, Hungary; 4 Department of Medical Physics and Informatics, University of Szeged, Szeged, Hungary

**Keywords:** Breast cancer, Oestrogen receptor, Progesterone receptor, Ki67, Reproducibility, ONEST

## Abstract

The reproducibility of assessing potential biomarkers is crucial for their implementation. ONEST (Observers Needed to Evaluate Subjective Tests) has been recently introduced as a new additive evaluation method for the assessment of reliability, by demonstrating how the number of observers impact on interobserver agreement. Oestrogen receptor (ER), progesterone receptor (PR), and Ki67 proliferation marker immunohistochemical stainings were assessed on 50 core needle biopsy and 50 excision samples from breast cancers by 9 pathologists according to daily practice. ER and PR statuses based on the percentages of stained nuclei were the most consistently assessed parameters (intraclass correlation coefficients, ICC 0.918–0.996), whereas Ki67 with 5 different theoretical or St Gallen Consensus Conference–proposed cut-off values demonstrated moderate to good reproducibility (ICC: 0.625–0.760). ONEST highlighted that consistent tests like ER and PR assessment needed only 2 or 3 observers for optimal evaluation of reproducibility, and the width between plots of the best and worst overall percent agreement values for 100 randomly selected permutations of observers was narrow. In contrast, with less consistently evaluated tests of Ki67 categorization, ONEST suggested at least 5 observers required for more trustful assessment of reliability, and the bandwidth of the best and worst plots was wider (up to 34% difference between two observers). ONEST has additional value to traditional calculations of the interobserver agreement by not only highlighting the number of observers needed to trustfully evaluate reproducibility but also by highlighting the rate of agreement with an increasing number of observers and disagreement between the better and worse ratings.

**Supplementary Information:**

The online version contains supplementary material available at 10.1007/s00428-021-03172-9.

## Introduction

Breast cancer is a heterogenous disease. This heterogeneity is reflected in the classifications of the disease along several parameters, e.g., histological type, imaging features, and several prognostic and/or predictive markers, some of which impact significantly on therapy.

Of the classifications, one of the most important is the segregation of carcinomas into oestrogen receptor (ER)–positive (ER+) and ER-negative (ER-) groups, of which only the first is likely to benefit from endocrine treatments. Currently, ER status is universally determined by immunohistochemistry (IHC) and the judgement of what constitutes an ER+ and ER- status is somewhat arbitrary and may depend on a number of pre-analytical and analytical issues, which are attempted to be minimalized by regularly updated guidelines such as the American Society of Clinical Oncology (ASCO) recommendations [1]. ER positivity had often been defined by an inclusive cut-off value of 10% [2, 3], then 1% [3]. At present, it is acknowledged that ER+ cancers with 1–10% ER expression may respond to endocrine treatment, but their response might be below expectations, and therefore, these tumours have been allocated to the category of low-ER-expressing carcinomas [1, 4]. Indeed, the level of ER expression reflects the degree of endocrine responsiveness as exemplified by the response to adjuvant tamoxifen therapy in the function of the Allred scores (derived sum of the intensity subscores 0–3 and semiquantitative percentage of positive cells subscores 0–5) [5]); the greater the score, the better the response [6].

Progesterone receptors (PR) also influence endocrine responsiveness. Earlier thought to reflect only the integrity of the ER pathway [4], recently they have been proposed to be actively involved in this pathway [7]. The evaluation of PR and its interpretation is similar to that of ER, and the Allred scoring is also applicable.

Ki67 is a protein which is expressed in variable amounts through the cell cycle, except in the G0 phase, and is a proliferation marker of prognostic significance [8]. Several cut-offs have been suggested to divide ER+ tumours into the low proliferation good prognosis (luminal A-like) category and the more aggressive, more proliferative (luminal B-like) one [9–12]. Despite the accepted prognostic role, owing to concerns about standardization, Ki67 is not part of general recommendations, although it is part of the IHC4 prognostic classifier [13]. As an estimate of proliferative tumour cells, it is also part of Hungarian guidelines for assessing breast carcinomas [14].

ER, PR, and Ki67 assessment by microscopy requires the quantification of nuclei that stain with the relevant antibodies. The common method of doing this is by eyeballing, i.e., having a look at the slide and estimating the amount of tumour cell staining. This may be tuned by estimating the area occupied by 100–200 cells, made more precise by counting 500–2000 cells [15], facilitating the count with an application [16, 17], or by using digital image analysis [18–20] or artificial intelligence [21]. Because of the costs and time required for the latter methods deemed more precise and reproducible, eyeballing is probably the most generally used method worldwide and is not obviously worse than some forms of digital image analysis [22].

Reproducibility issues have been analysed by multiple groups. In general, the interobserver agreement for ER and PR assessment for clinical management issues has been excellent for ER-negative cases and fair or good for strongly positive cases, with the worst consistency in allocating tumours to the moderate and low level of receptor positivity [23]. The interobserver consistency has most commonly been assessed by kappa statistics or intraclass correlation coefficients. We sought at investigating these predictive/prognostic tests by ONEST (Observers Needed to Evaluate Subjective Tests) [24].

**Fig. 1:**
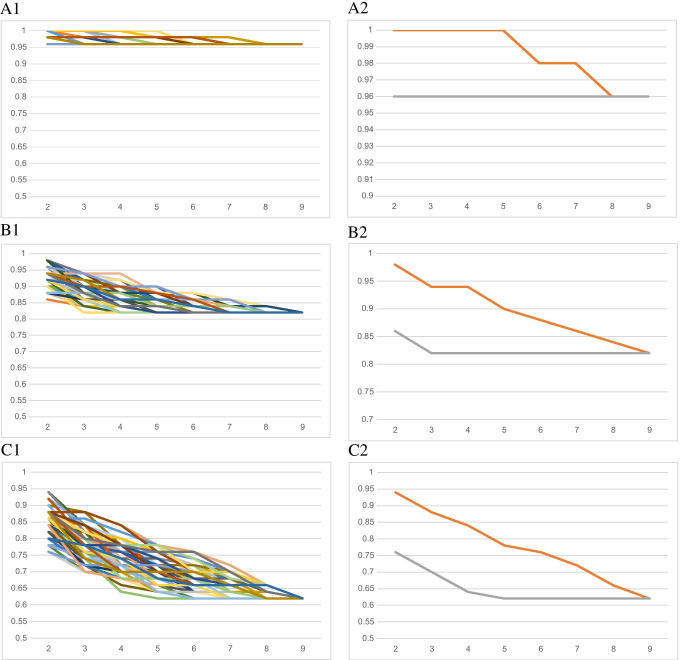
ONEST plots of ER (**A**), PR (**B**), and Ki67 (**C**) classifications into < 1%, 1–10%, and > 10% categories on CNB with all 100 random permutations of pathologists (A-B-C 1) and just the best and worst OPA values (A-B-C 2). Note: C2 demonstrates best that with the increasing number of pathologists, the OPA decreases till reaching a plateau with 4 pathologists. The classification can be characterized with the distance between the minimum and maximum OPA with 2 pathologists (0.94–0.76 = 0.18), the number of pathologists required for reaching the plateau (4), the approximate value of the plateau (0.64), and the OPA for all pathologists (0.62). Categorizations with good reproducibility have a narrow gap (bandwidth) between the maximum and minimum values, reach the plateau with few pathologists and have a high OPA with all pathologists (A1, A2). While A1, B1, and C1 demonstrate 100 OPAC each; A2, B2, and C2 show the minimum and maximum OPA values and do not necessarily overlap with an OPAC from the 100 permutations, but obviously overlap with an OPAC from all permutations. The worst scenario, i.e., the minimum OPA values were selected to characterize the categorizations

ONEST is a recently developed method to characterize how a subjective test requiring quantitative estimations of microscopic images can be reproduced by multiple observers. It has been created to analyse the performance of the atezolizumab related PD-L1 (programmed death-ligand 1) evaluation algorithm in breast cancer. More precisely, it was introduced to characterize how the estimation of the tumour area occupied by PD-L1 IHC stained immune cells being at least 1% (positive) or less (negative) could be reproduced by multiple observers. This PD-L1 assessment assay has been claimed to have 95% overall percent (proportion) agreement (OPA; i.e., the proportion of cases with full agreement on classification) on the basis of 2 observers [24], but empiricism suggested that the diagnostic test was less reproducible. ONEST is based on plotting the OPAs (0–1; corresponding to 0–100% agreement) against the increasing number of pathologists (observers) for 100 permutations randomly selected from all possible permutations of pathologists (i.e., the factorial product of the number of pathologists involved). Examples follow in the “Results” section, Fig. 1. Each plotted OPA for a given permutation results in an OPA curve (OPAC), and the 100 OPACs represent the full ONEST plot. The resulting ONEST plots highlight the number of pathologists where the OPA levels off, reaching a plateau. ONEST therefore suggests the number of pathologists required to reach this plateau (i.e., the number of observers to give adequate estimations of reproducibility); the plateauing value itself gives an estimate of overall agreement that can be expected. Finally, the graph also illustrates the OPA for all pathologists (the percentage of cases in which all raters agree) at the point to which all curves converge on the right side of the ONEST plot. It is also possible to visualize the greatest difference in agreement between two observers (wide versus narrow curve ranges). By applying ONEST to the PD-L1 algorithm tested, about 40% agreement plateau was reached with 8 observers [24]. Well reproducible tests have high values of OPA with low numbers of raters to reach the plateau and a small difference between the best and worst agreement of two raters.

This study was performed to evaluate the assessment of 3 IHC based biomarkers with nuclear staining by means of ONEST. The aims were to test the applicability of the new method in reproducibility studies, to see how ONEST can contribute to visualize discrepancies in classifications and to compare the 3 similarly evaluated biomarkers in terms of reproducibility and conduct with ONEST.

## Materials and methods

From the archives of the Bács-Kiskun County Teaching Hospital, 100 breast cancer cases with routine determination of ER, PR, and Ki67 were selected. The cases included 50 core biopsy samples which were taken with a policy to obtain at least 3 cores by 14-G needle biopsy gun (CNB) and 50 samples from unrelated resected tumour specimens (EXC). These cases were relatively consecutive, but some ER-PR- cases were discarded to allow better variation of the ER and PR values.

The IHC was performed with monoclonal antibodies 6F11 (Novocastra, Leica, Newcastle, UK) for ER, PgR312 (Novocastra, Leica, Newcastle, UK) for PR, and MIB1 (Dako-Agilent, Glostrup, Denmark) for Ki67. Participants were asked to report the percentage of cell staining for all three IHC reactions, along with the average staining intensity and Allred scores for ER and PR.

The ER and PR data were categorized as negative (< 1% staining), weekly positive (1–10%), and positive (> 10%). Mean intensity scores were given as nil (0), weak (1), medium (2), or strong (3). The Allred scores were categorized into broader groups (0, 2 vs 3–4 vs 5–6 vs 7–8), following the European Working Group for Breast Screening Pathology earlier practice [23].

The Ki67 values were assessed following the Hungarian breast pathology recommendations, which allow for eyeballing-based estimation of the Ki-67-labelling fraction with rounding to the closest 5%. Individual practice includes an estimation similar to ER and PR, but also more quantitative estimations like delineation of groups of about 100 cells and counting labelled cells in a few such sized groups. Five categorizations were evaluated: (1) with the same percentages as for ER and PR—although this has no practical value, it makes the results directly comparable with the steroid hormone receptor values; (2) with cut-offs suggested by the 2009 St Gallen consensus (i.e., ≤ 15%, 16–30%, and > 30% for low, intermediate, and high proliferation)[9]; (3) with a cut-off suggested by the 2011 St Gallen consensus (i.e., ≤ 13% and > 13% for low and high proliferation) [10]; (4) with a cut-off suggested by the 2013 St Gallen consensus (i.e., ≤ 20% and > 20% for low and high proliferation) [11]; and finally (5) with cut-offs suggested by the 2015 St Gallen consensus (i.e., at least 10% less than the median labelling of ER+ breast cancers for low labelling, at least 10% more than this median value for high proliferation, and the range in between for intermediate labelling) [12]. For this, the median Ki67 labelling (15%) of ER+ cases diagnosed in 2020 (*n* = 170) was used.

Rating reliability was analysed by the intraclass correlation coefficient (two-way random effects, absolute agreement, single rater/measurement; ICC (2, 1) [25]).

ONEST, as initially described by Reisenbichler et al. [24], was calculated for a randomly selected 100 permutations of the 362,880 (= 9!) possible permutations of ranked pathologists. The Kruskal Wallis test was applied to characterize and compare minimum values (i.e., the lowest plot—the “worst performance”); *p* values < 0.05 were considered statistically significant. The calculations were performed with the Real Statistics Add-Ins of Excel [26]. Details of the ONEST calculation are provided in Supplementary Material 1.

Since no patient data were used in this non-interventional retrospective study, no ethical approval was deemed necessary.

## Results

Nine pathologists, including 2 residents trained in breast pathology, have evaluated the 100 cases. They all had experience in the field of breast pathology, ranging from > 1 to > 25 years.

As the consistency of classifying the cases is dependent on the percentage of cells staining, with 0% and 100% being the easiest to categorize unanimously, Supplementary Fig. 1 demonstrates the boxplots for the main descriptive statistical features of the 50 CNB and 50 EXC specimens for the 3 nuclear markers assessed. As the cases were continuous but with the exclusion of some ER- cases, the median scores for the markers are only characteristic for the cases assessed; but to some extent, they also reflect breast cancer cases encountered in routine practice. The median percentage (interquartile range) of ER+ , PR+ , and Ki67+ cells as assessed by the 9 pathologists in biopsies vs excision specimens were 95 (30) vs 95 (15) (ER), 60 (89) vs 73 (95) (PR), and 20 (85) vs 10 (20) (Ki67), respectively. These values highlight that most nuclei stained for ER, less nuclei labelled with PR, and the least with Ki67.

The OPAs per diagnostic category are displayed in Supplementary Tables 1 and 2. The 100% agreement per diagnostic category for ER and PR was high in both CNB and EXC specimens (38 to 47/50 cases) but was somewhat worse for a similar distribution of Ki67 (31/50) on CNB and less than 50% (22/50) for Ki67 on EXC (Supplementary Table 1). With different St Gallen recommendations on interpreting Ki67 labelling values, consensus on categorization was best on CNB with the 2011 two-tiered-classification: 30/50 cases were classified with 100% agreement (Supplementary Table 2).

The ICC values for the evaluated parameters are shown in Table 1. According to these, most classifications relating to the ER and PR status of the tumours have an excellent or good to excellent level of reliability. In contrast, all Ki67 related classifications have moderate or moderate to good reliability. The difference in ICC values of the 3-category-based (1% and 10% cut-off) classification of ER or PR vs Ki67 is striking, whereas the difference in ICC values of different Ki67 categorization is less prominent. No major or consistent differences are seen in the ICC values of CNB and EXC specimens.

**Table. 1:**
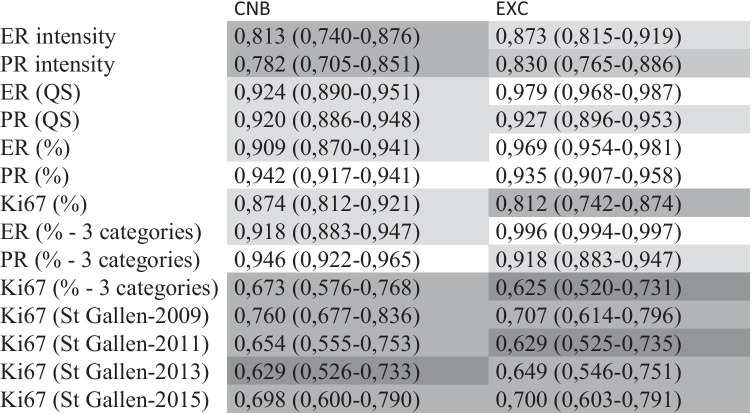
ICC (95% credible interval, CI) values for the investigated categories

*ER* oestrogen receptor, *PR* progesterone receptor, *QS* quick score or Allred score; intensity refers to average intensity scorings; (%) refers to the recorded percentage values with all different values representing a different category, 3 categories refer to < 1%, 1–10%, and > 10% categorization, St Gallen—year refers to the categories of low/(intermediate)/high Ki67 labelling as defined by the St Gallen Consensus Conference of the given year (see “Methods” section). The greyscale reflects the categorization of the level of reliability into excellent (ICC > 0.9), good to excellent, good (ICC > 0.75–0.9), moderate to good and moderate (ICC > 0.5–0.75) from white to deeper shades of grey; the 95% CIs are taken into account for the categorization [25]

As demonstrative examples, ONEST plots of the ER, PR, and Ki67 classifications of CNB samples reflected in Supplementary Table 1 (i.e., with categories < 1%, 1–10%, and > 10%) are shown in Fig. 1. The A1, B1, and C1 parts of the figure demonstrate OPACs of ER (A1), PR (B1), and Ki67 (C1) classifications of 100 randomly selected permutations of 9 pathologists, whereas only the minimum and maximum values of these OPA values are shown in the A2, B2, and C2 parts. Rather than demonstrating all possible ONEST plots, the minimum, maximum, and mean OPA values are shown in Supplementary Table 3, and the differences between the maximum and minimum OPAs, the OPA for all 9 pathologists, and the number of pathologists to reach the plateau are shown in Table 2.

**Table. 2:** Main results of the ONEST analyses of different parameters

	Maximum OPA differences	Pathologists needed for plateau	OPA with 9 pathologists
ER categories (< 1%, 1–10%, > 10%) CNB	0.04	2	0.96
ER categories (< 1%, 1–10%, > 10%) EXC	0.02	2	0.98
ER intensity CNB	0.32	5	0.48
ER intensity EXC	0.36	4	0.38
ER Allred scores (0,2; 3–4; 5–6; 7–8) CNB	0.12	4	0.72
ER Allred scores (0,2; 3–4; 5–6; 7–8) EXC	0.10	2	0.90
PR categories (< 1%, 1–10%, > 10%) CNB	0.12	3	0.82
PR categories (< 1%, 1–10%, > 10%) EXC	0.18	3	0.76
PR intensity CNB	0.36	4	0.38
PR intensity EXC	0.42	4	0.36
PR Allred scores (0,2; 3–4; 5–6; 7–8) CNB	0.22	5	0.48
PR Allred scores (0,2; 3–4; 5–6; 7–8) EXC	0.20	3	0.58
Ki67 categories (< 1%, 1–10%, > 10%) CNB	0.18	4	0.62
Ki67 categories (< 1%, 1–10%, > 10%) EXC	0.26	4	0.44
Ki67 St Gallen 2009 CNB	0.30	4	0.32
Ki67 St Gallen 2009 EXC	0.28	4	0.38
Ki67 St Gallen 2011 CNB	0.18	5	0.6
Ki67 St Gallen 2011 EXC	0.24	4	0.5
Ki67 St Gallen 2013 CNB	0.22	5	0.52
Ki67 St Gallen 2013 EXC	0.26	5	0.54
Ki67 St Gallen 2015 CNB	0.3	4	0.32
Ki67 St Gallen 2015 EXC	0.34	5	0.26

As concerns the classifications according to the 1% and 10% cut-offs or the different St Gallen criteria, the intensity scores for ER and PR, and the Allred scores lumped into 4 categories, there were no significant differences (Kruskal–Wallis tests *p* > 0.05) between CNB and EXC sample OPAs for the PR intensity scores and the Ki67 categories according to St Gallen 2013 criteria; all the other classifications significantly differed in OPAs for CNB and EXC specimens. Agreement was better on CNB specimens for ER intensity, PR status, Ki67 categories with 1% and 10% cut-offs, St Gallen 2011 and 2015 cut-offs and was better on EXC specimens for ER status, ER and PR Allred scores, and Ki67 classification according to St Gallen 2009.

Using the < 1%, 1–10%, and > 10% cut-offs for categorization, there were significant differences in the minimum (and average) OPA values from the ONEST plots between any pairs of ER, PR, and Ki67s both on CNB and EXC specimens.

The 4-category (0.2 vs 3–4, vs 5–6 vs 78) Allred score grouping minimum OPA values were also significantly different for ER and PR on both CNB and EXC specimens, whereas these values for the scores for average intensity of staining showed significant differences only for CNB specimens and not for EXC specimens (*p* = 0.44).

As concerns the classification of Ki-67 labelling indices into low vs high (vs intermediate if defined) proliferation according to different definitions proposed by consecutive St Gallen consensus conferences, the highest rate of OPA was noted with the 2013 proposal, i.e., a classification based on ≤ 20% vs 20%, and this was significantly better than any other St Gallen recommendation–based segregation. However, ICC values still suggested a moderate to good (CNB) or good (EXC) level of reliability (Table 1).

**Fig. 2:**
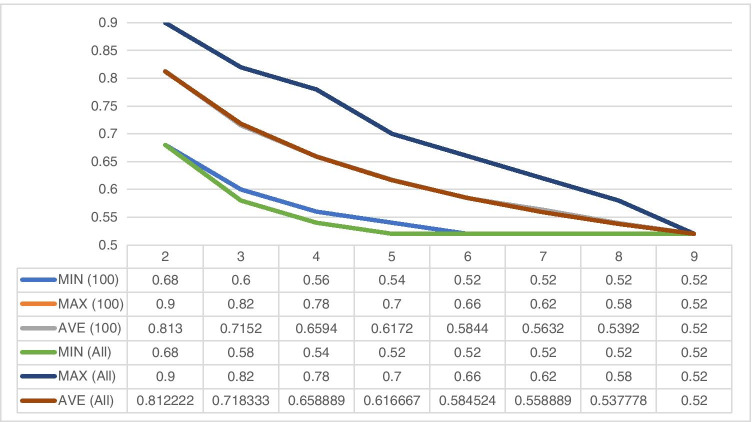
Comparison of OPAs derived from 100 and all permutations of pathologists for Ki67 categorization according to St Gallen 2013 recommendation. MIN: minimum, MAX: maximum, AVE: average, (100): for the 100 permutations, (All): for all 9! permutations. The MIN(All) and MAX(All) represent the worst and best OPAC, whereas the MIN(100) and MAX(100) curves lay on the worst and best OPA values and do not necessarily represent an OPAC. The AVE values are just derived from the 100 or 9! OPA values belonging to the number of pathologists on the x-axis. The MAX values (curves) overlap completely. The AVE curves virtually overlap completely and the MIN(100) vs MIN(All) curves deviate slightly, but the differences are not significant (*p* = 0.64; Kruskal Wallis)

As 9! (362,880) is still a manageable number, the minimum values of OPAs from the 100 random permutations were compared with the minimum values of OPAs from all permutations (i.e., the lowest OPAC). No significant differences were noted, most comparisons (Kruskal Wallis) yielded *p* = 1, and *p* values ranged from 0.64 to 1 (Fig. 2).

## Discussion

It is recognized that many factors influence the assessment of ER, PR, and Ki67 by IHC. This study concentrated on interpretational issues only, although two different types of material were evaluated in parallel: in contrast to whole section excision material, core biopsies have better fixation parameters and a smaller overall area to evaluate, potentially diminishing the discrepancies between observers.

With 100 cases mostly reflecting daily routine, ER and PR statuses (negative vs low positive vs positive) were the most reproducible with excellent or excellent to good classification of reliability (Tables 1 and 2). ONEST suggested that the categorization of ER showed the highest rates of OPA, and even 2 observers were sufficient to reflect reproducibility of assessment of the ER status, whereas PR was characterized by slightly lower OPA values and by 3 observers required for reflecting reproducibility (Tables 1 and 2, Fig. 1). The results suggest that these tests are valuable as assessed in daily practice. Although no recommendation exists to use Ki67 with < 1%, 1–10%, and > 10% categories, to allow better comparison with the determination of ER and PR, the virtual exercise of classifying cases according to these cut-offs was also done: the ICC suggested moderate or moderate to good reproducibility, the OPAs per increasing number of pathologists were lower, and the number of observers required for better assessment of reproducibility was 4 (Tables 1 and 2, Fig. 1). As all tests reflected the estimation of the percentage of stained tumour cell nuclei (without the influence of staining intensity) and their classification according to the same cut-off limits, the difference between the individual tests was only the proportion of stained nuclei and the size of the specimen (greater for EXC than CNB). It has been found in several studies that intermediate categories are less reproducible than categories at the extremes [23, 27, 28], and indeed, as indicated in the results (see also Supplementary Fig. 1), Ki67 staining proportions were often away from the extremes, which seems typical for this marker [29].

The intensity of staining was also assessed for ER and PR, and although the ICC values were reasonably good or even good to excellent (range 0.78–0.87), the ONEST analysis suggested that OPA values were low (0.36 to 0.48), with less than half of the pathologist agreeing, and therefore, 4 to 5 pathologists are needed to assess reproducibility. As the Allred quick scores are composed of subscores for intensity and for the proportion of stained cells, these consequently had ICC values reflecting excellent (with the 95% CI, good to excellent) reliability. However, the ONEST analysis of Allred scores reflected up to 22% difference between two observers, and 2 to 5 pathologists were required to assess reproducibility, with the worst results for PR assessment on CNBs (Table 2).

The comparison of ER, PR, and Ki67 with the 1% and 10% cut-offs suggested that the last biomarker was the least reproducible, and this could probably be explained by the relatively wide range of the stained cells per case. On the basis of daily practices reflected in this study, different classifications of low vs high (vs intermediate when defined) proliferation categories are not excellently reproducible (Table 1); the ICC values ranged from 0.63 to 0.76. Interestingly, the best ICC value was that of a 3-tiered classification (St Gallen 2009) [9] for CNB specimens. In keeping with the lower ICC values for any Ki67 determination (than for ER or PR staining), the ONEST analysis also suggested higher maximal differences between 2 observers (up to 34%), lower OPAs with all observers (26% as a minimum), and higher number of pathologists required to reflect reproducibility (mostly 5). The two-tiered systems of St Gallen recommendations from 2011 [10] and 2013 [11] had better parameters (lower maximum differences between 2 observers and higher OPAs for all observers).

It is evident from improved ICC values reported by the International Ki67 Working Group that scoring consistency of Ki67 can also be improved by standardized reporting, even without image analysis [16], and standardization is the way forward to achieve reliable Ki67 assessments. However, this study was not devised to increase reproducibility, but reproducibility was described as basic data, and the analysis was complemented by the newly developed ONEST method, to see what this can add to studies of reproducibility in case of biomarkers deemed suitable for prognostic or predictive conclusions. As hypothesized, ONEST can complement conventional statistics of agreement. It can prove or simply visualize that a biomarker is reliable, due to its easy assessment and natural distribution (like ER in our series; high plots with narrow bandwidth, Fig. 1A). It can also highlight weaknesses of biomarker assessment (high interrater differences, i.e., wide band between the top and the bottom curves, and low OPA values with all observers included, Fig. 1). This is in addition to the original aim of ONEST to determine the number of observers needed for the plot to reach a kind of plateau, i.e., the number minimally required to reliably reflect reproducibility. In this context, the results of some earlier reports, including one of ours [28], may be challenged on the basis of the number of observers involved; in the referred study, only three observers were included for the categorization of Ki67 staining according to the St Gallen 2009 criteria, whereas the current ONEST analysis would suggest at least 4, for reliable estimations.

Our results may also have an influence on current practice. While the eyeballing assessment of ER and PR staining and the determination of the Allred quick scores seem reliable, the same type of evaluation of Ki67 staining does not. Lower ICC values and poorer ONEST profiles independent of the cut-off values used by different recommendations point to a greater need for a more standardized assessment of Ki67, as proposed by the International Ki67 Working Group [30].

In summary, we have applied ONEST for characterizing the reproducibility of three biomarkers, all evaluated by estimating the proportion of immunostained nuclei on CNB and EXC specimens. The differences in reproducibility were mainly explained by the distribution of the stained nuclei around or away from the extremes (0% and 100%). ONEST gave useful supplementary information and its plots helped in visualizing the results. The minimum OPA values, the greatest difference in OPA for 2 pathologists and the OPA for all pathologists, are all reflected in ONEST plots.

## Supplementary information

Below is the link to the electronic supplementary material.

Supplementary file1 (DOCX 39 kb)

Supplementary file2 (DOCX 54 kb)

Supplementary file3 (DOCX 32 kb)

## Data Availability

The data on individual scores are available on reasonable request.
