# Supplementary material for: The additional value of ONEST (Observers Needed to Evaluate Subjective Tests) in assessing reproducibility of oestrogen receptor, progesterone receptor, and Ki67 classification in breast cancer
Source: Virchows Arch. 2021 Aug 20;479(6):1101–9. doi: 10.1007/s00428-021-03172-9 (PMC8724065; doi:10.1007/s00428-021-03172-9)
Supplement: Supplementary file 1 — Supplementary file1 (DOCX 39 kb) [file 428_2021_3172_MOESM1_ESM.docx]

Supplementary figure 1: Boxplots of ER, PR and Ki67 values as rated by the 9 observers in CNB (c1-c50) and EXC (c51-c100) cases

The boxes have an upper yellow part with the top representing the median value of the upper (2^nd^) half of the data (3^rd^ quartile, Q3), the lower grey part with the bottom representing the median value of the lower (1^st^) half of the data (1^st^ quartile, Q1) and the transition between the two parts representing the median value of all data. The box itself gives the interquartile range (IQR=Q3-Q1). The x symbols refer to the mathematical mean (average). Top whiskers refer to maximum and bottom whiskers to minimum values. For some cases, parts of the boxplot are overlapping, and therefore, are not visualized separately.

ER

PR

Ki67
