# Supplementary material for: The additional value of ONEST (Observers Needed to Evaluate Subjective Tests) in assessing reproducibility of oestrogen receptor, progesterone receptor, and Ki67 classification in breast cancer
Source: Virchows Arch. 2021 Aug 20;479(6):1101–9. doi: 10.1007/s00428-021-03172-9 (PMC8724065; doi:10.1007/s00428-021-03172-9)
Supplement: Supplementary file 2 — Supplementary file2 (DOCX 54 kb) [file 428_2021_3172_MOESM2_ESM.docx]

Supplementary Table 1

Agreement on the three-category-classification (positive, weakly positive and negative) of the steroid receptor statuses and analogous Ki67 distribution

|  | CNB | CNB | CNB | EXC | EXC | EXC |
| --- | --- | --- | --- | --- | --- | --- |
|  | ER status | PR status | Ki67 "status" | ER status | PR status | Ki67 "status" |
| Majority opinion: positive (>10%) |  |  |  |  |  |  |
| 100% agreement | 44 | 33 | 23 | 31 | 30 | 12 |
| <100% agreement (% range) | 3 (78-89%) | 2 (67-78%) | 11 (67-89%) | 1 (89%) | 2 (89%) | 11 (56-89%) |
| Majority opinion: low positive (1-10%) |  |  |  |  |  |  |
| 100% agreement | 0 | 1 | 8 | 0 | 0 | 10 |
| <100% agreement (% range) | 0 | 4 (56-78%) | 8 (56-89%) | 0 | 2+2x0.5 (44-89%)* | 17 (56-89%) |
| Majority opinion: negative (<1%) |  |  |  |  |  |  |
| 100% agreement | 3 | 7 | 0 | 8 | 8 | 0 |
| <100% agreement (% range) | 0 | 3 (89%) | 0 | 0 | 6+2x0.5 (44-89%)* | 0 |

CNB: core needle biopsy samples, EXC: excision samples; * There were 2 cases with equal (4 and 4) ratings into negative and low positive with an additional one into positive.

Supplementary Table 2

Agreement on the different Ki67 categorization according to consecutive St Gallen consensus conference recommendations

| CNB | St Gallen 2009 | St Gallen 2011 | St Gallen 2013 | St Gallen 2015 |
| --- | --- | --- | --- | --- |
| Cut-offs | (>30%, 16-30%, <=15%) | (>=14%, <14%) | (>=20%, <20%) | (>=25%, 6-24%, <=5%) |
| Ki67 High |  |  |  |  |
| 100% agreement | 5 | 22 | 10 | 12 |
| <100% agreement (% range) | 7 (66-89%) | 12 (67-89%) | 11 (56-89%) | 9 (56-89%) |
| Ki67 Intermediate |  |  |  |  |
| 100% agreement | 0 | NA | NA | 2 |
| <100% agreement (% range) | 13 (56-89%) | NA | NA | 19 (56-78%) |
| Ki67 Low |  |  |  |  |
| 100% agreement | 11 | 8 | 11 | 3 |
| <100% agreement (% range) | 14 (44-89%) | 8 (56-89%) | 13 (56-89%) | 5 (56-89%) |
| EXC |  |  |  |  |
| Ki67 High |  |  |  |  |
| 100% agreement | 1 | 12 | 9 | 5 |
| <100% agreement (% range) | 6 (56-89%) | 11 (67-89%) | 9 (56-89%) | 8 (67-89%) |
| Ki67 Intermediate |  |  |  |  |
| 100% agreement | 0 | NA | NA | 8 |
| <100% agreement (% range) | 10 (56-89%) | NA | NA | 24 (56-89%) |
| Ki67 Low |  |  |  |  |
| 100% agreement | 18 | 14 | 19 | 0 |
| <100% agreement (% range) | 15 (44-89%) | 13 (56-89%) | 13 (56-89%) | 5 (56-89%) |

NA: not applicable

Supplementary Table 3 – Minimum, maximum and mean of the OPAs for 100 random permutations of 9 pathologists

1. ER, PR and Ki67 (<1%, 1-10% and >10% categories) on CNB and EXC specimens

|  | Minimum | Maximum | Average | Minimum | Maximum | Average |
| --- | --- | --- | --- | --- | --- | --- |
| No of observers | ER CNB | |  | ER EXC | |  |
| 2 | 0.96 | 1.00 | 0.98 | 0.98 | 1.00 | 0.996 |
| 3 | 0.96 | 1.00 | 0.974 | 0.98 | 1.00 | 0.994 |
| 4 | 0.96 | 1.00 | 0.969 | 0.98 | 1.00 | 0.992 |
| 5 | 0.96 | 1.00 | 0.966 | 0.98 | 1.00 | 0.990 |
| 6 | 0.96 | 0.98 | 0.962 | 0.98 | 1.00 | 0.988 |
| 7 | 0.96 | 0.98 | 0.961 | 0.98 | 1.00 | 0.984 |
| 8 | 0.96 | 0.96 | 0.96 | 0.98 | 1.00 | 0.983 |
| 9 | 0.96 | 0.96 | 0.96 | 0.98 | 0.98 | 0.98 |
|  | PR CNB | |  | PR EXC | |  |
| 2 | 0.86 | 0.98 | 0.921 | 0.80 | 0.98 | 0.899 |
| 3 | 0.82 | 0.94 | 0.885 | 0.76 | 0.94 | 0.852 |
| 4 | 0.82 | 0.94 | 0.863 | 0.76 | 0..94 | 0.823 |
| 5 | 0.82 | 0.90 | 0.849 | 0.76 | 0.88 | 0.80 |
| 6 | 0.82 | 0.88 | 0.839 | 0.76 | 0.84 | 0.786 |
| 7 | 0.82 | 0.86 | 0.829 | 0.76 | 0.82 | 0.776 |
| 8 | 0.82 | 0.84 | 0.822 | 0.76 | 0.78 | 0.768 |
| 9 | 0.82 | 0.82 | 0.82 | 0.76 | 0.76 | 0.76 |
|  | Ki67 CNB | |  | Ki67 EXC |  |  |
| 2 | 0.76 | 0.94 | 0.855 | 0.64 | 0.90 | 0.798 |
| 3 | 0.70 | 0.88 | 0.78 | 0.52 | 0.82 | 0.686 |
| 4 | 0.64 | 0.84 | 0.734 | 0.48 | 0.74 | 0.616 |
| 5 | 0.62 | 0.78 | 0.701 | 0.44 | 0.70 | 0.567 |
| 6 | 0.62 | 0.76 | 0.676 | 0.44 | 0.66 | 0.533 |
| 7 | 0.62 | 0.72 | 0.655 | 0.44 | 0.60 | 0.502 |
| 8 | 0.62 | 0.66 | 0.638 | 0.44 | 0.52 | 0.469 |
| 9 | 0.62 | 0.62 | 0.62 | 0.44 | 0.44 | 0.44 |

1. ER and PR intensity and Allred scores for CNB and EXC specimens

|  | ER intensity (0-1-2-3) CNB | | | ER intensity (0-1-2-3) EXC | | |
| --- | --- | --- | --- | --- | --- | --- |
| Observers | MIN | MAX | AVERAGE | MIN | MAX | AVERAGE |
| 2 | 0.58 | 0.9 | 0.772 | 0.5 | 0.86 | 0.71 |
| 3 | 0.54 | 0.82 | 0.671 | 0.42 | 0.72 | 0.574 |
| 4 | 0.52 | 0.76 | 0.612 | 0.4 | 0.64 | 0.495 |
| 5 | 0.5 | 0.68 | 0.574 | 0.38 | 0.58 | 0.453 |
| 6 | 0.48 | 0.64 | 0.54 | 0.38 | 0.52 | 0.424 |
| 7 | 0.48 | 0.58 | 0.514 | 0.38 | 0.5 | 0.402 |
| 8 | 0.48 | 0.54 | 0.496 | 0.38 | 0.42 | 0.389 |
| 9 | 0.48 | 0.48 | 0.48 | 0.38 | 0.38 | 0.38 |
|  | ER Allred scores (0,2; 3-4; 5-6; 7-8) CNB | | | ER Allred scores (0,2; 3-4; 5-6; 7-8) EXC | | |
|  | MIN | MAX | AVERAGE | MIN | MAX | AVERAGE |
| 2 | 0.82 | 0.94 | 0.878 | 0.9 | 1 | 0.959 |
| 3 | 0.76 | 0.9 | 0.827 | 0.9 | 0.98 | 0.938 |
| 4 | 0.72 | 0.86 | 0.794 | 0.9 | 0.96 | 0.927 |
| 5 | 0.72 | 0.84 | 0.771 | 0.9 | 0.96 | 0.919 |
| 6 | 0.72 | 0.82 | 0.751 | 0.9 | 0.94 | 0.912 |
| 7 | 0.72 | 0.8 | 0.736 | 0.9 | 0.94 | 0.906 |
| 8 | 0.72 | 0.78 | 0.726 | 0.9 | 0.92 | 0.903 |
| 9 | 0.72 | 0.72 | 0.72 | 0.9 | 0.9 | 0.9 |
|  | PR intensity (0-1-2-3) CNB | | | PR intensity (0-1-2-3) EXC | | |
|  | MIN | MAX | AVERAGE | MIN | MAX | AVERAGE |
| 2 | 0.52 | 0.88 | 0.677 | 0.5 | 0.92 | 0.680 |
| 3 | 0.42 | 0.7 | 0.550 | 0.44 | 0.74 | 0.547 |
| 4 | 0.4 | 0.64 | 0.481 | 0.4 | 0.64 | 0.480 |
| 5 | 0.38 | 0.58 | 0.439 | 0.38 | 0.52 | 0.438 |
| 6 | 0.38 | 0.5 | 0.412 | 0.38 | 0.46 | 0.409 |
| 7 | 0.38 | 0.44 | 0.397 | 0.36 | 0.44 | 0.389 |
| 8 | 0.38 | 0.4 | 0.386 | 0.36 | 0.4 | 0.372 |
| 9 | 0.38 | 0.38 | 0.38 | 0.36 | 0.36 | 0.36 |
|  | PR Allred scores (0,2; 3-4; 5-6; 7-8) CNB | | | PR Allred scores (0,2; 3-4; 5-6; 7-8) EXC | | |
|  | MIN | MAX | AVERAGE | MIN | MAX | AVERAGE |
| 2 | 0.64 | 0.86 | 0.751 | 0.7 | 0.9 | 0.814 |
| 3 | 0.54 | 0.74 | 0.637 | 0.6 | 0.82 | 0.733 |
| 4 | 0.52 | 0.66 | 0.578 | 0.58 | 0.76 | 0.689 |
| 5 | 0.5 | 0.62 | 0.546 | 0.58 | 0.72 | 0.650 |
| 6 | 0.48 | 0.58 | 0.523 | 0.58 | 0.68 | 0.622 |
| 7 | 0.48 | 0.56 | 0.504 | 0.58 | 0.64 | 0.604 |
| 8 | 0.48 | 0.52 | 0.489 | 0.58 | 0.62 | 0.589 |
| 9 | 0.48 | 0.48 | 0.48 | 0.58 | 0.58 | 0.58 |

1. Ki67 values by different St Gallen recommendations in CNB and EXC specimens

|  | | Min | Max | Average | Min | Max | Average |
| --- | --- | --- | --- | --- | --- | --- | --- |
| Observers | Ki67 (as ER and PR: <1%, 1-10%, >10%) CNB | | | | Ki67 (as ER and PR: <1%, 1-10%, >10%) EXC | | |
| 2 | 0.76 | | 0.94 | 0.854 | 0.64 | 0.90 | 0.798 |
| 3 | 0.68 | | 0.88 | 0.783 | 0.52 | 0.82 | 0.686 |
| 4 | 0.64 | | 0.84 | 0.736 | 0.48 | 0.74 | 0.616 |
| 5 | 0.62 | | 0.80 | 0.702 | 0.44 | 0.70 | 0.567 |
| 6 | 0.62 | | 0.76 | 0.675 | 0.44 | 0.66 | 0.533 |
| 7 | 0.62 | | 0.72 | 0.653 | 0.44 | 0.60 | 0.502 |
| 8 | 0.62 | | 0.66 | 0.636 | 0.44 | 0.52 | 0.469 |
| 9 | 0.62 | | 0.62 | 0.62 | 0.44 | 0.44 | 0.44 |
|  | Ki67 (StGallen 2009: <16%,16-30%,>30%) CNB | | | | Ki67 (StGallen 2009: <16%,16-30%,>30%) EXC | | |
| 2 | 0.54 | | 0.84 | 0.717 | 0.6 | 0.88 | 0.738 |
| 3 | 0.38 | | 0.72 | 0.580 | 0.46 | 0.78 | 0.625 |
| 4 | 0.32 | | 0.64 | 0.498 | 0.4 | 0.66 | 0.552 |
| 5 | 0.32 | | 0.56 | 0.442 | 0.38 | 0.64 | 0.5 |
| 6 | 0.32 | | 0.48 | 0.398 | 0.38 | 0.6 | 0.466 |
| 7 | 0.32 | | 0.46 | 0.369 | 0.38 | 0.52 | 0.431 |
| 8 | 0.32 | | 0.38 | 0.338 | 0.38 | 0.46 | 0.407 |
| 9 | 0.32 | | 0.32 | 0.32 | 0.38 | 0.38 | 0.38 |
|  | Ki67 (StGallen 2011: <14%. >=14%) CNB | | | | Ki67 (StGallen 2011: <14%. >=14%) EXC | | |
| 2 | 0.76 | | 0.94 | 0.854 | 0.66 | 0.9 | 0.818 |
| 3 | 0.7 | | 0.88 | 0.776 | 0.56 | 0.84 | 0.719 |
| 4 | 0.64 | | 0.84 | 0.727 | 0.52 | 0.76 | 0.659 |
| 5 | 0.62 | | 0.78 | 0.692 | 0.5 | 0.74 | 0.614 |
| 6 | 0.6 | | 0.74 | 0.663 | 0.5 | 0.7 | 0.585 |
| 7 | 0.6 | | 0.7 | 0.640 | 0.5 | 0.66 | 0.558 |
| 8 | 0.6 | | 0.64 | 0.621 | 0.5 | 0.58 | 0.528 |
| 9 | 0.6 | | 0.6 | 0.6 | 0.5 | 0.5 | 0.5 |
|  | Ki67 (StGallen 2013: <20%. >=20%) CNB | | | | Ki67 (StGallen 2013: <20%. >=20%) EXC | | |
| 2 | 0.68 | | 0.9 | 0.813 | 0.66 | 0.92 | 0.833 |
| 3 | 0.6 | | 0.82 | 0.715 | 0.6 | 0.88 | 0.749 |
| 4 | 0.56 | | 0.78 | 0.659 | 0.6 | 0.8 | 0.696 |
| 5 | 0.54 | | 0.7 | 0.617 | 0.54 | 0.8 | 0.656 |
| 6 | 0.52 | | 0.66 | 0.584 | 0.54 | 0.74 | 0.625 |
| 7 | 0.52 | | 0.62 | 0.563 | 0.54 | 0.68 | 0.595 |
| 8 | 0.52 | | 0.58 | 0.539 | 0.54 | 0.6 | 0.569 |
| 9 | 0.52 | | 0.52 | 0.52 | 0.54 | 0.54 | 0.54 |
|  | Ki67 (StGallen 2015: <6%. 6-24%. >24%) CNB | | | | Ki67 (StGallen 2015: <6%. 6-24%. >24%) EXC | | |
| 2 | 0.54 | | 0.84 | 0.682 | 0.46 | 0.8 | 0.683 |
| 3 | 0.4 | | 0.66 | 0.542 | 0.36 | 0.66 | 0.525 |
| 4 | 0.34 | | 0.58 | 0.449 | 0.3 | 0.56 | 0.427 |
| 5 | 0.32 | | 0.54 | 0.401 | 0.26 | 0.5 | 0.370 |
| 6 | 0.32 | | 0.42 | 0.367 | 0.26 | 0.44 | 0.329 |
| 7 | 0.32 | | 0.4 | 0.349 | 0.26 | 0.36 | 0.299 |
| 8 | 0.32 | | 0.34 | 0.332 | 0.26 | 0.3 | 0.276 |
| 9 | 0.32 | | 0.32 | 0.32 | 0.26 | 0.26 | 0.26 |
