# Supplementary material for: The additional value of ONEST (Observers Needed to Evaluate Subjective Tests) in assessing reproducibility of oestrogen receptor, progesterone receptor, and Ki67 classification in breast cancer
Source: Virchows Arch. 2021 Aug 20;479(6):1101–9. doi: 10.1007/s00428-021-03172-9 (PMC8724065; doi:10.1007/s00428-021-03172-9)
Supplement: Supplementary file 3 — Supplementary file3 (DOCX 32 kb) [file 428_2021_3172_MOESM3_ESM.docx]

**Supplementary material 1 – ONEST calculation algorithm**

**Abbreviations:** OPA: overall percent agreement (the proportion of cases in which the specific observers agree about the classification); OPAC: OPA curve (OPA values for a given permutation of observers, represented as a curve of the OPAs as a function of the number of observers); ONEST: observers needed to evaluate subjective tests; ONEST plot: all 100 OPACs of the 100 random permutations of observers.

**Input:** Measurement matrix *M*, where the columns represent the observers, the rows represent the cases. The *M[c, r]* cell at the intersection of column *c* and row *r* store the value measured by observer *c* for case *r*.

**Output:** The collection of OPACs (i.e. the ONEST plot).

Let *C = { 1,…,rows(M) }* be the set of case indices, *S = { 1,…,columns(M) }* the set of observer indices.

1. Generate *N ≤ factorial(|S|)* distinct permutations of set *S*.

2. For each permutation *P* calculate the corresponding OPAC by assigning the OPA value to each element of *P* at index *I ≥ 2* as follows:

1. **function** OPA(M, C, S, P, I):

2. c := 0

3. **for** **each** i **in** C:

4. **for** **each** j **in** S **where** j < I:

5. **if** M[P[j], i] ≠ M[P[j + 1], i]:

6. **goto** 3 and continue with the next i

7. c := c + 1

8. **return** c / |C|

In *Step 1*, *N* distinct permutations of observer numbers are generated. In our case *|S|* equals 9, as there are nine pathologists participating in the assessment. This allows for 9! = 362 880 distinct permutations, out of which we select *N = 100*. These permutations define the one hundred OPACs of the ONEST plot.

In *Step 2* each OPAC is calculated based on its corresponding permutation. Each permutation represents a certain order of observers participating in the test, and each OPA at the *I*_th_ index of the OPAC shows the number of cases where the first *I* observers are in agreement relative to the total number of cases (*|C|*). The *I = 1* case can be skipped as a single observer is always in agreement with his- or herself (OPA can be considered 1 in this case).

As an example, let us consider permutation 123456789. For *I = 2* we are comparing the measurements of *Observer 1* and *Observer 2* over all the cases, and count the number of times they are in agreement. For *I = 3* we also include *Observer 3* and count the cases in which they all agree. If the permutation was 453216798, then for *I = 3* we would consider *Observer 4*, *5* and *3*.
